# Supplementary material for: The interactome of intact mitochondria by cross-linking mass spectrometry provides evidence for coexisting respiratory supercomplexes
Source: Mol Cell Proteomics. 2017 Dec 8;17(2):216–32. doi: 10.1074/mcp.RA117.000470 (PMC5795388; doi:10.1074/mcp.RA117.000470)
Supplement: Supplemental Data [file supp_RA117.000470_133922_0_supp_23978_fzffwf.zip › spectra_annotation/mito_spectra_annotation/456-1-4-1-35-1.pdf]

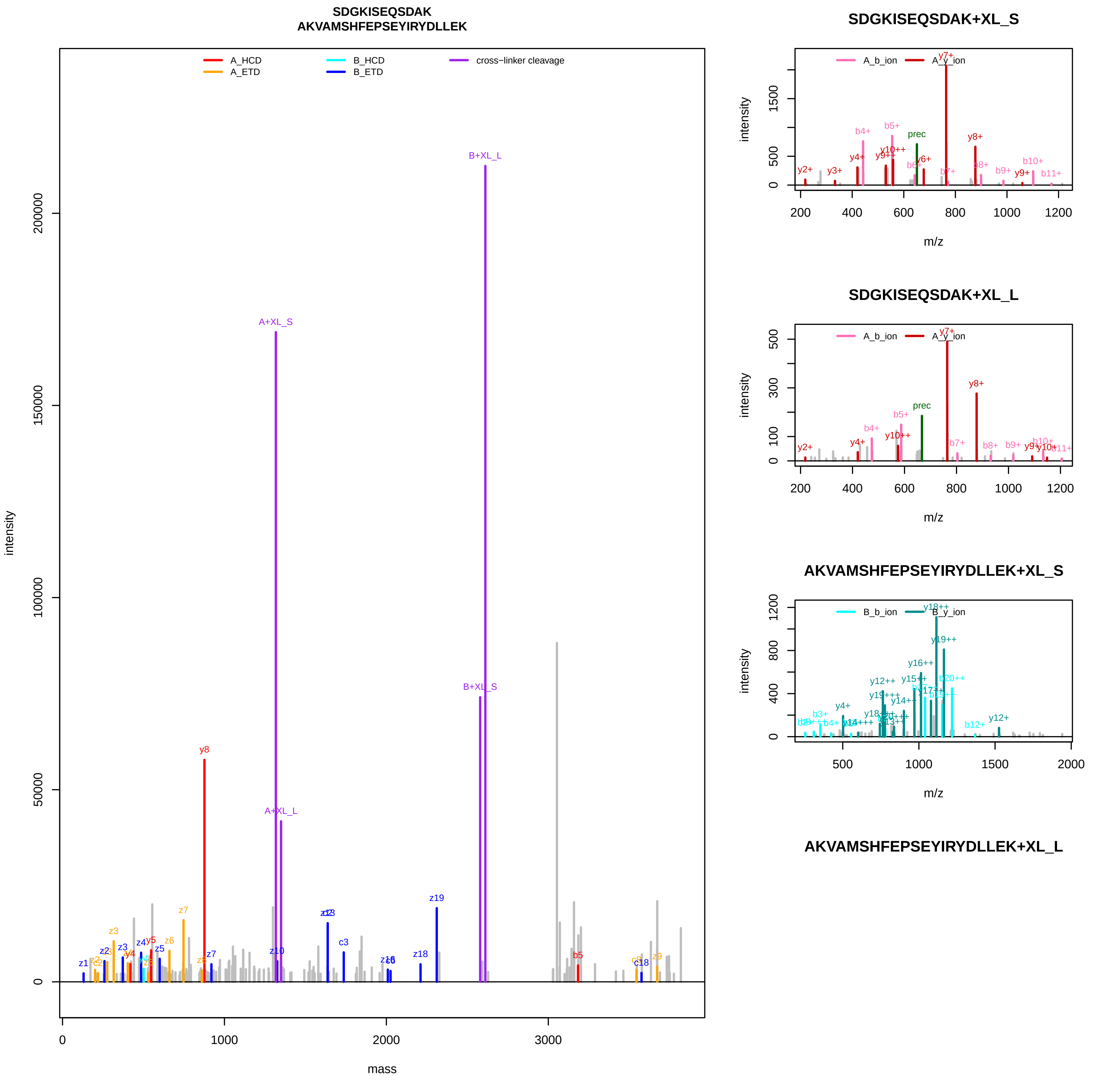

SDGKISEQSDAK
AKVAMSHFEPSEYIRYDLLEK
A_HCD
A_ETD
B_HCD
B_ETD
cross−linker cleavage
B+XL_L
A+XL_S
B+XL_S
A+XL_L
y8
z19
c3
z18
z7
z10
z6
z7
z3
z5
z4
y5
z2
z3
z1
b5
c18
z9
intensity
mass
SDGKISEQSDAK+XL_S
A_b_ion
A_y_ion
y7+
y8+
prec
b4+
b5+
y10++
y4+
y2+
y3+
b10+
b9+
y9+
b11+
m/z
SDGKISEQSDAK+XL_L
AKVAMSHFEPSEYIRYDLLEK+XL_S
B_b_ion
B_y_ion
y18++
y19++
y16++
y15++
y12++
b20++
y14++
y4+
b3+
y12+
b12+
AKVAMSHFEPSEYIRYDLLEK+XL_L
